# Pregnancy and Hereditary Neuropathy With Liability to Pressure Palsies: A Case Report and Narrative Review of the Literature

**DOI:** 10.7759/cureus.61236

**Published:** 2024-05-28

**Authors:** Vedha Priya Sudhakar, Jacquilin Sabu, Bethany Boatin, Karen Austin-Smith

**Affiliations:** 1 Department of Obstetrics and Gynaecology, University Hospitals of Leicester NHS Trust, Leicester, GBR; 2 Department of Obstetrics and Gynaecology, Kettering General Hospital, Kettering, GBR; 3 Department of Medicine, University of Leicester, Leicester, GBR

**Keywords:** hereditary neuropathy with liability to pressure palsy (hnpp), anesthetic complications, peripheral neuropathy, pregnancy, management of hnpp during pregnancy

## Abstract

Pregnancy in patients with hereditary neuropathy with liability to pressure palsy (HNPP) can present unique challenges. This is due to the potential exacerbation of neurological symptoms and the need for careful management during the antepartum, intrapartum, and postpartum periods. In this case report, we will discuss the successful management of a young pregnant female with a history of HNPP delivered by cesarean section. We will also review the existing literature on the management of pregnant patients with HNPP, focusing on the multidisciplinary input and strategies to minimize the risk of complications during labor and delivery. Reporting cases of pregnancy in HNPP is important for increasing awareness among clinicians and optimizing patient care.

## Introduction

Hereditary neuropathy with liability to pressure palsies (HNPP) is an autosomal dominant disorder of peripheral nerves. It is characterized by recurrent motor or sensory neuropathy following nerve compression, stretch, or trauma, affecting up to 16 per 100,000 individuals. HNPP is caused by the deletion of chromosome 17p11.2, resulting in a deficiency of peripheral myelin protein 22 (*PMP22*) [1]. While the clinical manifestations of HNPP vary widely, patients commonly experience weakness, sensory disturbances, and pain in the affected nerves, typically triggered by pressure or repetitive movements, with nearly 50% of episodes resulting in complete recovery within days to weeks [1,2]. The risk of inheritance to the child is 50% with variable penetrance [1]. Our case report outlines the management of pregnancy in a female with a known history of HNPP. In addition, we conducted a comprehensive literature review to guide the best practices in the management of pregnant females with HNPP.

## Case presentation

A 24-year-old pregnant female of White-British ethnicity (gravida 4, para 0, previous history of three first trimester miscarriages) was referred to our antenatal clinic at 27 weeks and three days due to her history of HNPP. At the age of 15 years, she was diagnosed with HNPP after developing peripheral paresis of the arm following prolonged writing at school. Diagnostic evaluation included electromyography and genetic testing, which revealed a deletion of chromosome 17 in the *PMP22* gene, confirming the diagnosis of HNPP. There was no history of HNPP in her family. During pregnancy, she occasionally experienced paresthesia in her lower limbs, especially toward the latter half of pregnancy, with no lasting neurological complications. She received care under the perinatal mental health team for anxiety. She had multiple attendances at triage for reduced fetal movements (RFM) and lower abdominal pain. At 38 weeks, due to recurrent RFM and an estimated fetal weight on the 10th centile, she was advised for delivery as per the local guidelines.

Antenatal multidisciplinary team input was sought from an obstetrician, anesthetist, neurologist, and pediatrician. Neurological intervention was not required in her case as she did not suffer from any palsy during pregnancy and the pediatric team suggested genetic testing may not be appropriate at birth unless the child is symptomatic. She opted to have an elective cesarean section at 38 weeks under spinal anesthesia for maternal request, recurrent episodes of reduced fetal movements, and small for gestational age baby.

She was scheduled for a neurological assessment in the pre-anesthetic clinic. If she opted for vaginal delivery in labor prior to her cesarean section, precautions were put in place to reduce the risk of neurological injury. This included avoiding prolonged positioning, especially in lithotomy, and refraining from performing instrumental delivery as it can pressurize and distend the pudendal, obturator, and femoral nerve in addition to the risk of neonatal palsy for the baby if affected by HNPP. We planned to avoid compression stockings and intermittent pneumatic compression devices intraoperatively during cesarean section, with meticulous attention to pressure points by using gel pads and pillows. She had an arterial line for blood pressure monitoring to avoid cuff pressure during the procedure and frequent re-positioning during the postoperative period along with the use of an air mattress. She received 10 days of thromboprophylaxis. Her postoperative period was uneventful, and she did not develop any neurological complications in her postpartum period. Genetic testing was not done on the baby, as it was asymptomatic.

## Discussion

Results

An electronic database search was conducted from Medline (1946 to September 2023), Emcare (1974 to September 2023), UpToDate, Embase (1995 to September 2023), and Google Scholar. Our narrative literature review focused on the management of pregnancy in females with HNPP. We identified 12 articles from the literature search, and eight case reports focusing on pregnancy and HNPP were included as shown in Table 1 [3-10].

**Table 1:** Review of management of pregnant females with HNPP from published case reports HNPP: hereditary neuropathy with liability to pressure palsy

Study	Year	Known diagnosis of HNPP	Gestational age at delivery (in weeks + days)	Mode of delivery/type of anesthesia if used	Presentation	Final conclusion
Bolger and Stewart [3]	2019	Yes	38 + 6	Vaginal delivery/epidural, analgesia	Winging of scapula following prolonged flight as the initial presentation. Common peroneal nerve palsy following epidural analgesia in previous pregnancy	Necessity for awareness among clinicians on history and symptoms that may identify patients with HNPP. Careful anesthetic considerations for the avoidance of nerve injury.
Samuel et al. [4]	2019	Yes	36	Elective cesarean section for obstetric cholestasis/spinal anesthesia	Tongue paresis with previous use of general anesthesia, temporary loss of arm sensation for two days postpartum from the cuff pressure used for non-invasive blood pressure monitoring	Use an arterial line to monitor blood pressure and avoid compression stockings and pneumatic calf compression devices perioperatively.
Soumpasis and Rolfsson [5]	2018	Yes	37 + 6	Emergency cesarean section for preeclampsia/spinal anesthesia	Numbness diffusely distributed to hands and arms, mainly after night's sleep	Antenatal multidisciplinary team input is essential, prolonged immobilization should be avoided, and epidural and spinal anesthesia can be safely administered. Dense epidural regimens should be avoided, and nitrous oxide is contraindicated due to the potential neurotoxic effects.
Chilvers and Salman [6]	2011	No	Unknown	Semi-elective cesarean section for failed induction of labor/spinal anesthesia	Numbness over the back and abdomen 19 days postoperatively	Accurate history-taking, senior review, and early neurological assessment are important.
Peters and Hinds [7]	2005	No	Unknown	Emergency cesarean section/epidural anesthesia	Foot drop in immediate postpartum	Seek neurological review in a history suggestive of recurrent entrapment neuropathies.
Berdai and Benhamou [8]	2004	Yes	38 + 3	Emergency cesarean section for preeclampsia/epidural anesthesia	No reports of neurological symptoms during pregnancy or postpartum, and the paper reports successful management of subsequent pregnancy by elective cesarean section/spinal anesthesia	Consult a neurologist to conduct a neurological assessment. Avoid prolonged immobilization during work. Avoid nerve compressions and stretching. Avoid dense sensory/motor block. Consider cesarean section.
Lepski and Alderson [9]	2001	Yes	42	Vaginal delivery/epidural analgesia	Foot numbness following prolonged kneeling	Involve a neurologist and an anesthetist in the antenatal period, conduct a neurological assessment, avoid prolonged immobilization in labor, avoid instrumental delivery, less dense epidural blockade, and consider cesarean section if pressure palsy arises during labor.
Molloy et al. [10]	2000	No	34	Vaginal delivery of twin pregnancy/no analgesia	Immediate bilateral wrist drop following delivery due to hand placement under knees for support during labor	Avoid sustained postures. Consider the use of oxytocin to reduce the chances of the patient developing a nerve injury.

Discussion

HNPP is an underestimated disease; hence, more reporting of pregnancies in HNPP cases is important for increasing awareness among clinicians in addition to multidisciplinary team input for proper counseling and management [11]. HNPP should be considered as a differential diagnosis for antepartum or postpartum neuropathy following minor injury or prolonged compression [7]. There is no effective standard treatment for HNPP, and management is primarily focused on addressing the symptoms.

Neurological assessment of the patient's baseline function and monitoring for any exacerbation of symptoms during pregnancy is recommended. During labor and delivery, strategies to minimize the risk of nerve compression and injury are essential. Depending on the position adopted during labor and contributing factors such as macrosomia or the use of forceps, there is a significant risk of nerve injuries, including the lateral femoral cutaneous nerve, femoral nerve, obturator nerve, common peroneal nerve, and sciatic nerve [12]. Thus, soft padding, frequent change of positions in labor, and avoiding the use of lithotomy stirrups and elastic straps for fetal monitoring are advised. Elective cesarean section may be considered in patients with HNPP, especially if there are concerns about prolonged labor including the need for induction of labor or instrumental delivery. In our case, an elective cesarean section was performed for maternal request following a multidisciplinary review. A retrospective study conducted in the United Kingdom on pregnant females with HNPP and *PMP22* gene-related neuropathies found no significant increase in antepartum or intrapartum complications in these cohorts. In addition, the type of delivery did not differ significantly from the normal population [4,11].

Anesthetic management is considered carefully in patients with HNPP, as certain medications and techniques may exacerbate neurological symptoms. Regional anesthesia, such as spinal or epidural anesthesia, is preferred over general anesthesia to minimize the risk of nerve injury such as tongue paresthesia [4,9]. Bolger and Stewart [3] reported the precautions necessary for successful epidural analgesia in their article. Careful patient positioning, avoidance of prolonged nerve compression with non-invasive blood pressure monitoring, soft padding, utilization of the lowest effective analgesia concentration in intermittent doses, and ultrasound-guided peripheral nerve blockade are the recommended measures to prevent symptom exacerbation. Nitrous oxide use is contraindicated because of its potential neurotoxic effects especially when used in patients at risk of neuropathies [5]. Conversely, literature indicating the use of oxytocin to reduce the risk of nerve injuries is considered inappropriate to be generalized to all cases [10].

**Figure 1:**
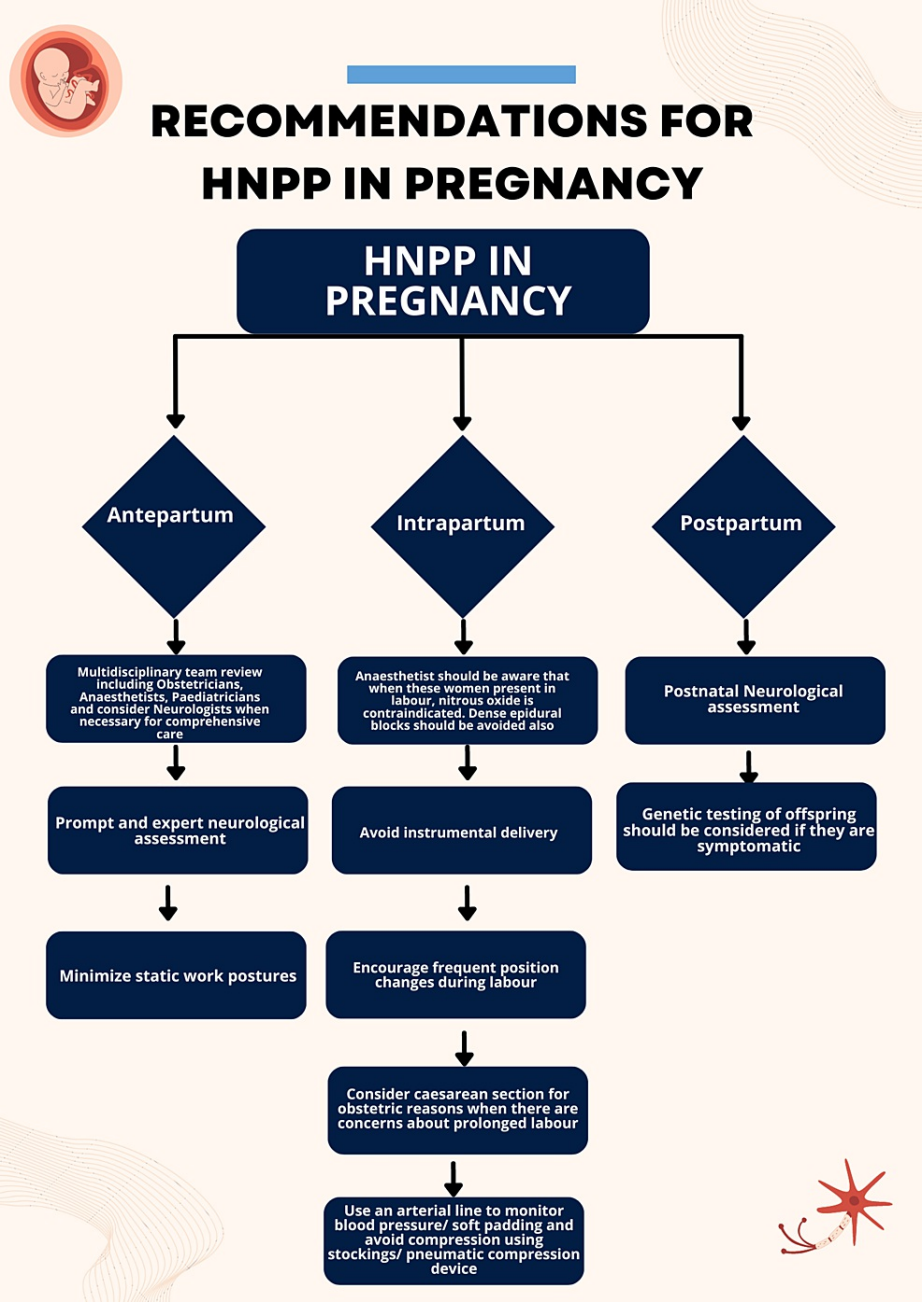
Practical recommendations for managing HNPP patients during pregnancy HNPP: hereditary neuropathy with liability to pressure palsy Image credit: This image was created by the authors of this article.

Genetic counseling is an important aspect of the management of pregnancy in patients with HNPP, as there is a 50% risk of inheritance for offspring. Prenatal testing and pre-implantation genetic diagnosis are not routinely recommended. While genetic testing may not be necessary at birth unless the child is symptomatic, parents should be informed about the risk of HNPP and the potential implications for future pregnancies [1]. Figure 1 summarizes the practical recommendations for managing HNPP patients during pregnancy.

## Conclusions

In conclusion, pregnancy in patients with hereditary neuropathy with liability to pressure palsy presents unique challenges requiring a multidisciplinary approach and careful coordination of care. Neurological assessment, judicious decision-making regarding the mode of delivery balancing patients' expectations, precautions to avoid nerve compressions, and careful anesthetic considerations are essential components of management. While there is limited literature on this topic, each reported case contributes valuable insights into the optimal care of these patients. Future research should focus on larger cohort studies to better understand the natural history of pregnancy in patients with HNPP and further refine management guidelines.

## References

[1] van Paassen BW, van der Kooi AJ, van Spaendonck-Zwarts KY, Verhamme C, Baas F, de Visser M (2014). PMP22 related neuropathies: Charcot-Marie-Tooth disease type 1A and hereditary neuropathy with liability to pressure palsies. Orphanet J Rare Dis.

[2] Sessa M, Nemni R, Quattrini A, Del Carro U, Wrabetz L, Canal N (1997). Atypical hereditary neuropathy with liability to pressure palsies (HNPP): the value of direct DNA diagnosis. J Med Genet.

[3] Bolger AA, Stewart PA (2019). Anesthetic considerations of hereditary neuropathy with liability to pressure palsies in an obstetric patient: a case report. A A Pract.

[4] Samuel K, Mead K, Cominos T, Weale N (2019). Spinal anaesthesia for elective caesarean section in a patient with hereditary neuropathy with liability to pressure palsies. Int J Obstet Anesth.

[5] Soumpasis I, Rolfsson H (2019). Parturient with hereditary neuropathy with liability to pressure palsy. Abstracts for the 2019 Scandinavian Society of Anaesthesiology and Intensive Care Medicine Congress in Copenhagen.

[6] Chilvers RJ, Salman MM (2011). Hereditary neuropathy with a liability to pressure palsies presenting as a case of sensory neuropathy following spinal anaesthesia for caesarean delivery. Int J Obstet Anesth.

[7] Peters G, Hinds NP (2005). Inherited neuropathy can cause postpartum foot drop. Anesth Analg.

[8] Berdai S, Benhamou D (2004). [Regional anaesthesia for labor adn delivery in a parturient with neuropathy with liability to pressure palsy (tomaculous neuropathy)]. Ann Fr Anesth Reanim.

[9] Lepski GR, Alderson JD (2001). Epidural analgesia in labour for a patient with hereditary neuropathy with liability to pressure palsy. Int J Obstet Anesth.

[10] Molloy FM, Raynor EM, Rutkove SB (2000). Maternal bilateral radial neuropathy during childbirth in hereditary neuropathy with a predisposition to pressure palsies (HNPP). J Clin Neuromuscul Dis.

[11] Skorupinska M, Ramdharry G, Byrne B, Laurá M, Reilly MM (2023). Pregnancy and delivery in patients with Charcot-Marie-Tooth disease and related disorders. Obstet Med.

[12] Mcdonald A (2008). Obstetrical nerve injury. https://mncyn.ca/wp-content/uploads/2013/08/volume31.pdf.

